# An organ-specific view on non-host resistance

**DOI:** 10.3389/fpls.2015.00526

**Published:** 2015-07-20

**Authors:** Roxana Strugala, Rhoda Delventhal, Ulrich Schaffrath

**Affiliations:** RiBa-Lab, Department of Plant Physiology (Biology III), Rheinisch-Westfälische Technische Hochschule Aachen University, Aachen, Germany

**Keywords:** *Arabidopsis*, *Magnaporthe oryzae*, rice blast, roots, spikes, wheat

## Abstract

Non-host resistance (NHR) is the resistance of plants to a plethora of non-adapted pathogens and is considered as one of the most robust resistance mechanisms of plants. Studies have shown that the efficiency of resistance in general and NHR in particular could vary in different plant organs, thus pointing to tissue-specific determinants. This was exemplified by research on host and non-host interactions of the fungal plant pathogen *Magnaporthe oryzae* with rice and *Arabidopsis*, respectively. Thus, rice roots were shown to be impaired in resistance to *M. oryzae* isolates to which leaves of the same rice cultivar are highly resistant. Moreover, roots of *Arabidopsis* are also accessible to penetration by *M. oryzae* while leaves of this non-host plant cannot be infected. We addressed the question whether or not other plant tissues such as the reproductive system also differ in NHR compared to leaves. Inoculation experiments on wheat with different *Magnaporthe* species forming either a host or non-host type of interaction revealed that NHR was as effective on spikes as on leaves. This finding might pave the way for combatting *M. oryzae* disease on wheat spikes which has become a serious threat especially in South America.

On an evolutionary scale current plant pathogen interactions can be seen as the outcome of a process in which heterotrophic organisms tried to get access to energy-containing organic molecules produced by photoautotrophic plants. This struggle for life, of course, had great ecological impact and strongly affected structure and dynamics of plant communities (Gilbert, 2002). It can be speculated that plant pathogens evolved from saprophytes feeding on dead plant tissue and then, in a process of constant adaptation, expanded their life-style and acquired capabilities to subsist also on living plants. In this regard, it is still a matter of debate whether in ancient times land plants were initially resistant to occurring microbes and disease evolved as an exception (Heath, 1991). In natural habitats only a very limited number of microbes is able to colonize a given plant species and to cause severe disease symptoms while being unable to be pathogenic on the majority of other plant species to which they are not adapted. The latter phenomenon is known as non-host resistance (NHR) and believed to be the most common form of plant resistance occurring in the wild (Heath, 2000). Because NHR is effective to a plethora of potential pathogens and durable in natural ecosystems, NHR has become an intensely studied field of research aiming at translating these capabilities to elite crop plants. At current, there are a few examples in which the transfer of a single dominant gene from a non-host to a host plant confers resistance to host pathogens (for review see Bettgenhaeuser et al., 2014). However, usually NHR is seen as a complex, quantitative phenomenon relying on several genes. According to recent review articles these may either be stacked *R*-genes, whose products recognize different pathogen effectors, or genes contributing to the general pathogen recognition and defense associated with PAMP-triggered immunity (Schweizer, 2007; Schulze-Lefert and Panstruga, 2011; Senthil-Kumar and Mysore, 2013; Stam et al., 2014). Therefore approaches targeting only single genes might be too simplistic. The present perspective article is written to broaden our view on NHR and might provide novel considerations for translational research.

## Smooth Transitions Between Host and Non-host Status of Plants

Non-host resistance operates at the species level, which means that all genotypes of a plant species are resistant to all genotypes of a pathogen species (Heath, 1991). However, this definition brings about the intrinsic problem of experimentally testing all genotypes of both interaction partners against each other. Under laboratory conditions we have to limit the number of genotypes investigated, and this restriction in turn might lead to false estimations. However, this is not a serious problem: if only a single pathogen isolate was overlooked while the vast majority of other members of the same pathogen species was unable to cause disease it is still well-founded to talk about a non-host interaction. The same holds true when looking at the plant side: if only a single genotype of a plant species could be attacked by a given pathogen that is otherwise incapable of reproducing on other genotypes of this plant species, this would not give the plant host status. The latter scenario was experimentally traced by showing that mutations in crucial NHR genes can result in a transition from a non-host to a host plant (Lipka et al., 2008). An example for that are the *Arabidopsis thaliana PEN* (*PENETRATION*) genes. Mutations in each of three *PEN*-loci made *Arabidopsis* more accessible to non-host pathogens such as *Blumeria graminis* f.sp. *hordei* (*Bgh*, barley powdery mildew; Collins et al., 2003; Lipka et al., 2005; Stein et al., 2006), *Phakopsora pachyrhizi* (Loehrer et al., 2008), or *Magnaporthe oryzae* (Nakao et al., 2011; Schreiber et al., 2011). However, only combined mutations in gene loci compromising penetration- and post-penetration defense mechanisms rendered *Arabidopsis* fully susceptible to *Bgh* (Lipka et al., 2005). Despite this single susceptible genotype, *Arabidopsis* remains a non-host for *Bgh*. This would be further supported by the argument, that *Arabidopsis* genotypes with negated NHR are artificially generated mutants which would not have occurred in nature. However, there is also the example of a barley cultivar with diminished NHR to *Magnaporthe* which was not particularly selected for a break in NHR. Generally, barley is a host for *M. oryzae*, the causal agent of rice blast disease, but a non-host for *Magnaporthe* species isolated from other grass genera such as *Digitaria* or *Pennisetum* (Zellerhoff et al., 2006). Recently, it was reported that the barley cultivar Nigrate showed susceptibility to a few isolates of the *Digitaria*-infecting *M. grisea* species otherwise non-adapted to barley (Hyon et al., 2012; Nga et al., 2012). Thus, Nigrate may represent a natural occurring barley genotype with compromised NHR to *M. grisea*. Nevertheless, the majority of barley genotypes remain non-hosts for *M. grisea*. From the above mentioned considerations, it can be concluded that genetic differences between host and non-host plant species might be small, and loss of function in some NHR genes may result in a continuous transition from a definite non-host toward a real host plant. This process of acquiring or losing host vs. non-host status was also addressed in a review of Niks and Marcel (2009).

## More to be Considered in Non-host Resistance: Organ-specificity

**FIGURE 1:**
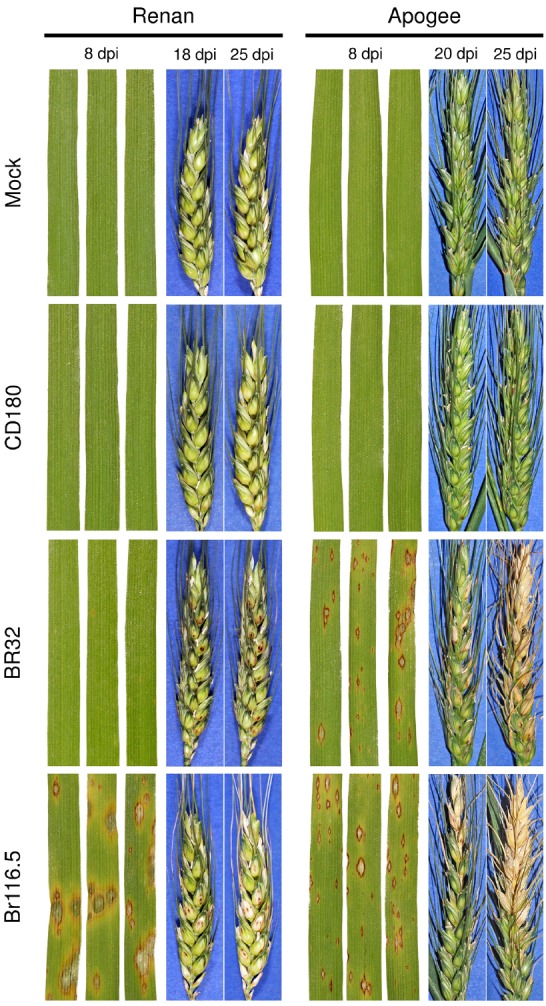
**Leaf and spike infection phenotypes of different wheat cultivars in response to inoculation with *Magnaporthe* host and non-host isolates.** Wheat cultivars Renan and Apogee were grown under controlled conditions in a phytotron. Primary leaves were inoculated 8 days after sowing with a spore suspension of different *Magnaporthe* isolates (BR32 and Br116.5 both belong to *M. oryzae* whereas CD180 was isolated from a *Pennisetum* host plant) at a density of 200,000 conidia mL^–1^ or a mock solution without spores. Spikes of both cultivars were similarly treated at flowering stage. Disease scoring was done for leaves at 8 days post inoculation (dpi) and for spikes at two time points as indicated to monitor the progress of disease. (Renan was kindly provided by Lesley Boyd, John Innes Centre, Norwich, UK; Apogee was received from Eckhard Koch, JKI Darmstadt, Germany; Br116.5 from Yukio Tosa, Kobe University, Japan; BR32 and CD180 were obtained from CIRAD Montpellier, France).

The previous paragraph dealt with the observation that NHR to a particular pathogen could be compromised in some genotypes of a plant species without putting the whole concept into question. A further, so far underestimated, facet to be considered when talking about resistance is the phenomenon of organ-specificity. Plants are composed of different organs or tissues: (i) roots, (ii) shoots, (iii) leaves, and (iv) the reproductive system and all of them are targets for plant pathogens. While some pathogens are specialists, attacking and reproducing only on a single tissue, e.g., *Claviceps purpurea* causing ergot on young grass ovaries (Tudzynski and Scheffer, 2004), others are less strict and attack both below- and above-ground plant organs. An example for the latter is *Hyaloperonospora arabidopsidis*, the downy mildew pathogen of *Arabidopsis* (Mauch-Mani and Slusarenko, 1993). This oomycete overwinters with oospores in decaying plant material on the ground. After germination the pathogen infects roots from which it proliferates into shoots and leaves where it finally produces conidia. These conidia are dispersed by wind and in nature infect above-ground parts of *Arabidopsis* plants. However, when artificially inoculated onto roots, conidia are also capable of infecting this plant organ. Hermanns et al. (2003) elucidated in one of the earlier investigations in this field that roots of *Arabidopsis* genotypes are susceptible to isolates of *H. arabidopsidis* to which leaves of the same genotype are resistant. Because essential genes necessary for resistance and signaling in leaves were shown to be also expressed in roots, it was concluded that organ-specific properties may alter the effectiveness of host resistance. Going back in time, it was already proposed by Heath (1991) that defense mechanisms in roots and aerial plant parts may differ. Later on, the work of Jansen et al. (2006) added to this emerging field reporting on ineffective host resistance expressed by rice roots against a *M. oryzae* isolate that was avirulent on leaves of the same rice cultivar. The authors further boosted the importance of organ-specificity by showing that even the phenomenon of acquired resistance did not work with the same efficacy in rice roots as observed for rice leaves. Because *M. oryzae* has relatives, namely *Gaeumannomyces graminis* or *M. rhizophila*, which are pathogenic on plant roots, it was speculated *M. oryzae* might have kept the ability to infect roots from a common ancestor although this might not be of relevance in current natural systems (Sesma and Osbourn, 2004). Balmer et al. (2013) reported that roots may even display a stronger defense response than leaves as evidenced for maize challenged with the shoot infecting pathogen *Colletotrichum graminicola*. Collectively, these reports demonstrate that resistance of plants to pathogens underlies organ-specific determinants. What about NHR? Schreiber et al. (2011) addressed this question using the non-host interaction between the plant model organism *Arabidopsis* and the fungal pathogen model organism *M. oryzae*. As anticipated for a non-host interaction, leaves of different *Arabidopsis* wild type accessions were resistant to the pathogen. By contrast, *Arabidopsis* roots of all tested genotypes were susceptible to *M. oryzae* displaying invasive growth similar to foliar infection of host plants. Though, the fungus failed to cross the border between root and hypocotyl tissue. Thereby, the authors demonstrated that, at least for this host-pathogen combination, NHR can be impaired in particular plant organs. This was a novel finding, challenging the attractive strategy of using NHR for the improvement of crop plants. It could be argued, though, that root infection of a strictly above-ground living pathogen is artificial and has no relevance for agriculture. However, this leads to the question of NHR efficacy in other aerial plant tissues, i.e., the reproductive system. We investigated this using the pathosystem wheat and *M. oryzae*. While it is known from rice that *M. oryzae* predominantly infects leaves and the panicle neck (Talbot, 1995), the fungus attacks spikes of barley and wheat leading to a severe disease phenotype referred to as head blast (Maciel et al., 2013). The latter is an emerging disease of increasing importance especially in South America and neither resistant cultivars nor effective fungicides are available (Castroagudín et al., 2014). Therefore, we tested the efficacy of NHR in wheat spikes to different *Magnaporthe* species. For this assay we selected the French winter bread wheat cultivar Renan which has, e.g., a durable type of resistance against stripe rust (Dedryver et al., 2009) and the cultivar Apogee which was specifically developed in cooperation with the National Aeronautics and Space Administration in the US and has a very short time until flowering which makes it especially interesting for work with flower infecting pathogens (Mackintosh et al., 2006). Both wheat cultivars were inoculated with two host isolates (*M. oryzae* Br116.5 and BR32) and a non-host isolate recently renamed as *Pyricularia penniseticola* (CD180, Klaubauf et al., 2014). As shown in Figure 1, leaves of the wheat cultivars Renan and Apogee are susceptible to *M. oryzae* isolate Br116.5 as evidenced by typical blast lesions occurring 8 days after inoculation. While leaves of cultivar Apogee were also susceptible to *M. oryzae* isolate BR32, leaves of cultivar Renan, by contrast, expressed partial resistance to this isolate which is in accordance with previous reports (Tufan et al., 2009). Disease severity of spike infection in response to the host isolate was comparable to that of leaves from the same cultivar. Both wheat cultivars form a non-host type of interaction on leaves with *Magnaporthe* isolate CD180 derived from *Pennisetum* as shown similarly for barley (Zellerhoff et al., 2006). Importantly, spikes of both wheat cultivars were also resistant to isolate CD180, suggesting equally effective NHR mechanisms in leaves and the reproductive system. In further studies, however, it has to be determined whether or not underlying cellular defense responses or activation of non-host responsive genes are similar in both tissues. Thereafter, the use of constitutive or inducible promotors driving expression of essential NHR genes in spikes might open the route to improve wheat resistance to virulent *M. oryzae* isolates.

## Outlook

Despite the above mentioned considerations addressing the possible break-down of NHR in particular genotypes, the general concept of this broad-acting plant defense mechanisms still provides an attractive basis for translational research at durable resistance of crop plants. This long term goal can be achieved either by providing breeders with markers for selection of plant genotypes with a favorable combination of quantitative trait loci involved in NHR or by applying transgenic technologies aiming at the execution of NHR-mechanisms against host pathogens. During investigations of NHR phenomena, it has to be kept in mind that different plant organs might behave with graded responses to host and non-host pathogens. Therefore, each plant organ of interest has to be tested separately for its response to a given pathogen and it is not advisable to conclude, e.g., from a standard leaf-infection assay to root or flower disease phenotypes.

### Conflict of Interest Statement

The authors declare that the research was conducted in the absence of any commercial or financial relationships that could be construed as a potential conflict of interest.
